# Shedding light on participant selection bias in Ecological Momentary Assessment (EMA) studies: Findings from an internet panel study

**DOI:** 10.1371/journal.pone.0282591

**Published:** 2023-03-09

**Authors:** Arthur A. Stone, Stefan Schneider, Joshua M. Smyth, Doerte U. Junghaenel, Cheng Wen, Mick P. Couper, Sarah Goldstein

**Affiliations:** 1 Department of Psychology, University of Southern California, Los Angeles, CA, United States of America; 2 Dornsife Center for Self-Report Science, University of Southern California, Los Angeles, CA, United States of America; 3 Department of Biobehavioral Health, The Pennsylvania State University, State College, PA, United States of America; 4 Institute for Social Research, University of Michigan, Ann Arbor, MI, United States of America; Cambridge Health Alliance, UNITED STATES

## Abstract

Although the potential for participant selection bias is readily acknowledged in the momentary data collection literature, very little is known about uptake rates in these studies or about differences in the people that participate versus those who do not. This study analyzed data from an existing Internet panel of older people (age 50 and greater) who were offered participation into a momentary study (n = 3,169), which made it possible to compute uptake and to compare many characteristics of participation status. Momentary studies present participants with brief surveys multiple times a day over several days; these surveys ask about immediate or recent experiences. A 29.1% uptake rate was observed when all respondents were considered, whereas a 39.2% uptake rate was found when individuals who did not have eligible smartphones (necessary for ambulatory data collection) were eliminated from the analyses. Taking into account the participation rate for being in this Internet panel, we estimate uptake rates for the general population to be about 5%. A consistent pattern of differences emerged between those who accepted the invitation to participate versus those who did not (in univariate analyses): participants were more likely to be female, younger, have higher income, have higher levels of education, rate their health as better, be employed, not be retired, not be disabled, have better self-rated computer skills, and to have participated in more prior Internet surveys (all p < .0026). Many variables were not associated with uptake including race, big five personality scores, and subjective well-being. For several of the predictors, the magnitude of the effects on uptake was substantial. These results indicate the possibility that, depending upon the associations being investigated, person selection bias could be present in momentary data collection studies.

## Introduction

Under many circumstances, the ability to make population inferences is an important issue in studies that employ momentary assessment, such as EMA [1, 2] and the Experience Sampling Method [3]. In brief, EMA comprises a family of techniques that signal participants several times a day to complete brief surveys (1–5 minutes, usually) on their smartphones or similar device. It has been used to study many constructs, among those studied are affect, pain, symptoms, consumption, craving, social interactions, stress, the work environment, and the physical environment [4]. A central feature of EMA is that it allows researchers to study processes and relationships that occur at a moment-to-moment, within-person level as people go about their everyday activities. When it can be safely assumed that the investigated process is the same across all types of people, then representation of the population may not be important. However, this is hardly ever the case, and EMA research is very interested in the extent to which the processes or relationships differ for different groups of people in the population (for example, the relationship between social interactions and affect), where the observed relationship may not hold in a different group or population.

Concerns about the representativity of subjects–whether college students undergoing experiments for course credit or volunteers in clinical trials–have been raised in a number of different fields. An early, influential paper in psychology [5] discussed concerns about such bias, leading to Henrich, et al. [6] proposing an acronym (WEIRD, or Western, Educated, Industrialized, Rich, and Democratic) to describe the societies from which most subjects in psychology are drawn. Similarly, early work in economics (see [7]) has demonstrated that selection bias affects not only descriptive statistics (e.g., population means and proportions), but also more complex models (correlations, regression coefficients, structural equation models, etc.). Arguments for considering both internal and external validity have also been made in fields as diverse as program evaluation [8], epidemiology [9], and neuroscience [10]. Lavrakas, Traugott [11] provide additional examples (see also [12]).

Given the importance of sample selection for valid inferences, we turn to issues of participant selection bias in EMA research. As with all methods, momentary assessment methods have the potential for incurring significant participant selection bias; such concerns may be pronounced in circumstances where the demands of participation are high (e.g., many responses needed over the course of a day [13]). Although momentary research methods were first developed over 40 years ago, to our knowledge there is no systematic information available about participant selection bias in EMA studies or about where in the recruitment process nonparticipation occurs.

### Why would we expect selection bias in EMA studies?

One factor that may be central to determining who participates in momentary studies is the amount of perceived burden of the study protocol. That is, completing even brief surveys several times a day for a week requires considerable effort and may be intrusive when prompts occur at inconvenient moments. The notion that burden is generally associated with selection bias is not a new topic of investigation. A classic paper on burden was published by Bradburn [14], where he conceptualized burden in terms of a typical dyadic interchange and lamented the absence of practical solutions to reduce burden. Additional research on participant burden resulted from governmental actions designed to put a cap on burden (number of minutes) for governmental data collection activities [15] with the goal of increasing survey response rates and reducing selection bias.

Broadly speaking, momentary studies are relatively burdensome for many participants when compared with less involved studies, which often have lower time commitments. The degree of burden associated with a study design is surely determined by many factors pertaining to the rationale for the study, the participants studied, and the particular design features of study such as frequency of prompting and number of study days [13, 16]. A typical momentary study would likely have moderate to high burden, because it involves somewhat intrusive sampling in everyday life over a substantial period. However, the negative impact of burden on selection may be offset by other study features, such as when the purpose of a study is deemed relevant to one’s interests or future well-being or when it is of special interest to participants (e.g., investigations of disease processes and treatments in patients [17]).

There are other factors that may be of importance to participant selection in momentary studies. EMA protocols typically involve data collection with smartphones that run specialized programs or with handheld devices used exclusively for momentary data collection. Recent studies have discussed barriers to participation for such mobile data collection [18, 19], which shares some data collection features with EMA. For instance, having a requirement that participants download and install EMA programs may make the study less attractive or even preclude some people from participating (e.g., those who do not possess the technology required for a study), reducing study uptake. Conversely, those individuals familiar with technology may be more comfortable with EMA protocols.

The goal of this study was to increase our knowledge about potential selection bias in momentary studies by conducting secondary analyses of an Internet panel study that solicited members to participate in a one-week momentary study. To the best of our knowledge, this is the first report about EMA selection bias that has extensive data available about both the individuals agreeing to participate *and those who did not*. The reason for this is that the individuals who were invited to participate were drawn from a pre-existing probability panel whose members completed self-report surveys at regular intervals. The latter point is a key design feature of the current study, because it is almost always the case in EMA research that no or very little person-level information is available about nonparticipants. Since nothing is known about nonparticipants, it is impossible to directly compare the characteristics of nonparticipants to participants and understand how they may differ from each other. The downside of this particular sample is the reduced generalizability of the results to other settings and participants, which we discuss later.

### What do we know about factors associated with participant selection bias from prior research?

The selection of plausible predictors of participation versus non-participation in the current study (referred to as “selection variables”) was based on a review of variables related to participation in research studies of all types. Several studies examined sociodemographic and personality predictors of participation. Most prior literature has found that women were more likely to participate in studies than men [20–24]. Higher socioeconomic status, being employed, being married, and having higher education were positively associated with participation rates [20–23, 25]. Results for other demographic predictors, however, have often been mixed. For example, although some studies have found greater participation rates for older compared to younger adults [20, 26–28], others have found the opposite effect [29–31]. Specifically for momentary studies that require smartphones or similar technology, older people may have lower access to the technology than younger people (see https://www.pewresearch.org/internet/fact-sheet/mobile/).

Prior research also suggested that some ethnic and minority status characteristics are predictive of participation [22, 30]. Some studies have found that people who were non-Asian [32], white [30, 33, 34], or Western [35] were more likely to participate. Overall, however, the evidence here is also mixed [21]. Regarding personality, being social and more active has been associated with greater participation rates [26]. Some research has shown that people who respond to an online study invitation score higher on personality measures of agreeableness and openness compared to non-responders [36]. Similarly, active non-responders—people who directly express their unwillingness to participate—have been found to be less agreeable and less conscientious compared to responders [37]. Other research has shown that lower levels of openness and higher levels of conscientiousness predicted greater completion of follow-up surveys in a longitudinal study design [38].

Another line of prior research examined participant technology use, skill level, and motivational factors for predicting study uptake, which we thought would be particularly relevant for EMA studies. Owning a tablet and smartphone are both associated with increased likelihood to participate in studies [26, 39]. Higher self-rated smartphone skill levels are also related to greater participation rates [19, 39]. One study found that the specific brand of smartphone owned was a relevant predictor of study response in that owners of iPhone and HTC devices were more open to downloading a smartphone research application than owners of other smartphone brands [26]. Regarding motivational factors, frequent engagement in research studies has been generally related to better uptake rates [40] as has greater intrinsic respondent interest in the particular research topic that is under study [21]. In summary, prior non-EMA studies have provided rich (although often mixed) evidence of factors associated with selectivity of study samples. Here, we investigate whether or not many of these factors contribute to selection bias in EMA research.

### The present study

In the context of a well-characterized denominator (those who were offered participation) and across multiple points of potential exclusion and/or disinterest decision points, our goal in this research was to examine (a) overall rates of study uptake (participation rate, that is, those who consent to participate in the study) and (b) selection bias (associated with the selection variables). As mentioned above, this was a secondary analysis of an EMA study conducted within a pre-existing Internet panel, the Understanding America Study (UAS) panel (https://uasdata.usc.edu/). Thus, being offered participation in the EMA study was conditional upon participation in the UAS panel study. Within this context, there are various ways to define non-participation, which impacts both the estimated overall uptake rates and comparisons of participants versus non-participants on the selection variables.

Our first primary question was if all panel members who agreed to be in the momentary study (participants) were different from all who did not participate, regardless of the reason for nonparticipation. Uptake rate was computed by dividing the number of participants by the number of participants plus the number of nonparticipants (as defined). For many researchers, these statistics may be the most relevant for determining a study’s uptake rate and for examining possible selection bias.

The second primary question was similar to the first in that it compared participants with all nonparticipants, but it omitted from analyses nonparticipants without smartphones compatible with the UAS’s data collection software (an eligibility criterion). The rationale for this comparison was that eligibility criteria for the UAS study were specific to this study and eligibility criteria could be very different in other studies. Importantly, we do not know how many of the individuals excluded by the UAS eligibility criteria would have participated had they been given the opportunity, for example, if they had been provided with the smartphones required for the UAS momentary software program. Therefore, the overall uptake rate and selection variable associations are likely affected by these somewhat arbitrary and study-specific criteria.

One exploratory question supplements the findings from the primary questions. The question directly compares those who explicitly declined participation (i.e., excluding those who merely did not respond to the invitation in addition to those who were ineligible) with those who participated. Our expectation was that selection factors pertaining to the demands of the momentary study might be highlighted as the comparison focuses on the behavior of actively rejecting the momentary study.

Although not the primary focus of this study, we conducted two additional exploratory analyses that focused on the group of individuals who participated (consented) and examined predictors of whether or not they actually entered the EMA protocol, that is, provided any amount of EMA data and, separately, whether or not they completed at least half of the scheduled prompts. This information extends the reach of these analyses beyond consenting and provides additional information about possible selection bias regarding those who actually participated in the EMA protocol.

## Method

### Participants and procedures

The Understanding America Study (UAS) is a probability-based Internet panel that longitudinally tracks a sample of approximately 10,000 U.S. residents (https://uasdata.usc.edu/). Members are recruited exclusively through address-based sampling and respondents without prior access to the Internet receive a tablet and broadband access (but not smartphones), which ensures that the sample achieves wide coverage in populations typically underrepresented in opt-in or volunteer online panels [41]. About 12 percent of those invited to join the panel become panel members. This protocol was approved by the University of Southern California Institutional Review Board (approval number UP-14-00148) and written consent was obtained.

### Inviting panelists to participate in the EMA burst

The EMA data collections conducted in the UAS began in February, 2020, and consisted of 1-week of intensive repeated assessments in panel members 50 years of age or older (a primary focus of the UAS is on health and retirement). The bursts consist of momentary assessments of mood, pain, stress, and social relationships collected 6x/day along with other assessments (e.g., end-of-day daily diaries and voice recordings of participants describing the day’s occurrences). Invitations to fill out the EMA consent survey were made available in batches to UAS panelists (see S1 Appendix for text of email invitation). For each batch of invitations, the UAS system made an online screening survey available through the panelists’ profiles. Within 3 days of the study invitations, the study administrator followed up with an email to encourage participation. The first email described the study as an upcoming UAS research project focusing on health and activities. A link to the online screening survey was included in the email. Additional reminder emails were sent to those panelists who did not respond to the study invitation, spaced approximately one week apart after the initial invitation. Nonresponsive panelists were re-contacted again at the next batch of recruitment for the burst. A total participant payment of up to $100 was offered. For the analyses presented in this paper, we only considered panelists’ response until February 2021.

The screening survey asked panelists about their eligibility, contained information about the details of study participation, and informed panelists about the compensation associated with study participation. The primary inclusion criteria were having a smartphone with Android or iOS operating system (no minimum level of the operating systems was required) and with a voice and data plan billed on a monthly basis. There were two study protocols that differed in the following ways. The first protocol, which was used through April 3^rd^, 2020, included the use a wrist-worn accelerometer throughout the burst. The second protocol, which started May 18^th^, 2020, mentioned the accelerometry, but said it was temporarily suspended due to the novel coronavirus pandemic. In addition, in the second protocol participants were asked to provide brief voice recordings twice during the burst. These design components add burden to the study beyond the momentary assessments and should be taken into account when considering the resulting uptake rates. At the end of the study description (with either version), panelists were given the option of “Yes, I am interested in participating,” “No, I am not interested in participating,” and “Not sure, I need more information to decide.”

Those who expressed interest in participating were then provided with detailed instructions on installing the study application on their phone and were informed that the study team would contact them with more information regarding participation. Although no specific operating systems were part of the inclusion criteria, in fact, Android 5.0 or iOS 10.3 were the lowest level of the operating systems required for installation of the app. For those who indicated that they were not interested in participating, the survey asked for the reasons for the lack of interest. For those who indicated uncertainty in their interest in participating, additional descriptive material about the study was displayed on the follow up page in the screening survey. If the panelists still were unsure, then a research team member contacted the panelists to provide further details and to enroll eligible panelists who expressed interest in participating in the study.

## Materials

Fortunately, many of the concepts emerging from the literature review were assessed in the questionnaires that most UAS members had taken prior to being invited into the momentary study. We limited our analyses to demographic variables and specific constructs that emerged from the review.

### Demographic variables

Demographic information of all the UAS panelists was collected after they enrolled as a panelist and were re-assessed quarterly to ensure the information is up to date. Panelists were prevented from enrolling in additional UAS studies if the demographic profiles were last updated by the panelists more than 3 months ago. The demographic variables included in this analysis were from the latest update prior to when the invitation to participate in the EMA study was made available to the panelists.

### Prior participation in UAS surveys

The number of prior UAS surveys a respondent reported participating in was assessed in the same end-of-year questionnaire that assessed panelists’ self-reported computer skill level. Panelists were asked “Around how many surveys do you think you have taken with the Understanding America Study in the last year?” with the response options of “1”, “2–4”, “5–7”, and “8 or more.”

### Self-reported computer skill level

Panelists’ self-reported computer skill levels were assessed using two items through an end-of-year survey administered to UAS panelists from December, 2019 through February, 2020. Panelists who indicated that they currently own or use a desktop, laptop, or tablet computer received two questions: 1) “How would you rate your computer skill level” (response options “Beginner” “Moderate”, “Competent” to “Expert”) and 2) “In general, how confident are you in using a computer for writing tasks that involve typing on the computer keyboard such as answering email?” (response options “Not confident at all”, “Somewhat confident”, “Very confident”, “Completely confident”). These variables were measured on a subset (n = 2479) of the sample, the Ns for the analyses differ.

### Well-being and health

Life satisfaction was assessed with the question “Overall, how satisfied are you with your life?” (1–5 scale) and with the Cantril Ladder item, in which respondents are asked how they would rate their life on an imaginary ladder from 0 (worst possible) to 10 (best possible). Self-reported global health was assessed with two very similar questions “In general, how would you rate your overall health?” and “In general, would you say your health is?” which were both rated with response scales ranging from 1 (Excellent) to 5 (Poor). Some respondents had only one of the questions and that response was used; for those who were administered both questions, responses to the first question were used. Both the Cantril Ladder and Global Health variables were administered only to a third to half of the respondents (as shown in Table 1).

**Table 1:** Logistic regressions for each selection variable.

	Question 1 Comparing those who participated versus those who did not					Question 2 Comparing those who participated versus those who did not *omitting those who were ineligible for the study (incompatible phone)*				
Selection Variable	N	Uptake Rate	Relative to 29.1% Overall Uptake	Odds Ratio	Significance	N	Uptake Rate	Relative to 39.2% Overall Uptake	Odds Ratio	Significance
Invitation Month	3014				X^2^(4) = 6.0	2241				X^2^(4) = 7.5
February	476	33.2%	+4.1	ref		365	43.3%	+4.1	ref	
March	237	31.6%	+2.5	.932		171	43.9%	+4.7	1.024	
May	636	27.8%	-1.3	.776		457	38.7%	-0.5	.828	
August	622	28.3%	-0.8	.794		442	39.8%	+0.6	.867	
October	1043	27.9%	-1.2	.779		806	36.1%	-3.1	.740	
Age	3009				X^2^(5) = 72.1***	2237				X^2^(5) = 54.5***
< 55 years	604	35.8%	+6.7	ref		485	44.5%	+5.3	ref	
55–59 years	607	34.9%	+5.8	.959		470	44.9%	+5.7	1.015	
60–64 years	572	30.9%	+1.8	.800		418	42.1%	+2.9	.906	
65–69 years	529	27.7%	-1.4	.687		386	38.1%	-1.1	.767	
70–74 years	373	24.0%	-5.1	.565		267	33.7%	-5.5	.632	
> = 75 years	323	11.6%	-17.5	.235		211	17.9%	-21.3	.269	
Gender	3012			X^2^(1) = 26.6***		2240				X^2^(2) = 24.3***
Female	1641	33.0%	+3.9	ref		1239	43.7*%*	+4.5	ref	
Male	1371	24.4%	-4.7	.655		1001	33.5%	-5.7	.647	
Married	3011				X^2^(1) = 6.6	2239				X^2^(2) = .0
No	1212	26.5%	-2.6	ref		823	39.1%	-0.1	ref	
Yes	1799	30.9%	+1.8	1.238		1416	39.2%	+0.0	1.008	
Education	3012				X^2^(2) = 51.3***	2240				X^2^(2) = 20.9***
LT High school	173	14.5%	-14.6	ref		91	27.8%	-11.4	ref	
High school	513	19.4%	-9.7	1.426		333	29.8%	-9.4	1.105	
College	2326	32.4%	+3.3	2.836		1816	41.4%	+2.2	1.845	
Income	3000				X^2^(3) = 104.2***	2233				X^2^(3) = 23.0***
<$30,000	739	18.2%	-10.9	ref		423	31.7%	-7.5	ref	
$30,000–59,9999	806	25.1%	-4.0	1.505		564	36.1%	-3.1	1.214	
$60,000–99,999	735	32.3%	+3.2	2.137		583	40.6%	+1.4	1.474	
>$100,000	720	41.8%	+12.7	3.226		663	45.3%	+6.1	1.787	
Hispanic/Latino	3012			X^2^(1) = .0		2044				X^2^(2) = 1.74
No	2763	29.2%	+0.1	ref		2034	39.6%	+0.4	ref	
Yes	249	28.6%	-0.5	.974		206	34.8%	-4.4	.815	
Race	3014			X^2^(2) = 5.9		2241				X^2^(2) = 5.0
White	2465	29.9%	+0.8	ref		1840	40.1%	+0.9	ref	
Black	229	22.7%	-6.4	.686		163	31.8%	-7.4	.696	
Other	320	27.3%	-1.8	.877		238	36.7%		.864	
Working	3009			X^2^(1) = 47.1***		2237				X^2^(2) = 10.2***
No	1582	23.7%	-5.4	ref		1053	35.7%	-3.5	ref	
Yes	1427	35.1%	+6.0	1.746		1184	42.3%	+3.1	1.321	
Retired	3009			X^2^(1) = 23.2***		2237				X^2^(2) = 12.8***
No	1828	32.3%	+3.2	ref		1404	42.0%	+2.8		
Yes	1181	24.1%	-5.0	.665		833	34.4%	-4.8	.722	
Disabled	3009			X^2^(1) = 18.3***		2237				X^2^(2) = 1.4
No	2644	30.4%	+1.3	ref		2037	39.5%	+0.3	ref	
Yes	365	19.5%	-9.6	.552		200	35.3%	-3.9	.834	
Computer keyboard skills	2360			X^2^(3) = 137.8***		1706				X^2^(3) = 77.5***
Not confident at all	114	7.6%	-21.5	ref		55	15.4%	-23.8	ref	
Somewhat confident	592	19.1%	-10.0	2.892		358	31.6%	-7.6	2.561	
Very confident	800	33.4%	+4.3	6.151		594	45.0%	+5.8	4.560	
Completely confident	854	46.2%	+17.1	10.539		699	56.4%	+17.2	7.253	
Computer confidence	2360			X^2^(3) = 109.5***		1706				X^2^(3) = 65.4***
Beginner	243	11.0%	-18.1	ref		123	21.8%	-17.4	ref	
Moderate	906	26.7%	-2.4	2.956		628	38.4%	-0.8	2.254	
Competent	1018	42.1%	+13.0	5.920		796	53.8%	+14.6	4.240	
Expert	193	45.0%	+15.9	6.686		159	54.9%	+15.7	4.442	
Prior UAS Surveys	2359			X^2^(2) = 14.8***		1705				X^2^(2) = 20.6***
Lowest & Low	39	7.8%	-21.3	ref		26	11.6%	-27.6	ref	
High	281	27.2%	-1.9	4.465		214	35.8%	-3.4	4.260	
Highest	2039	34.5%	+5.4	6.294		1465	48.0%	+8.8	7.092	
Extraversion	3007				X^2^(3) = 8.5	2237				X^2^(3) = 2.4
1^st^ Quartile	744	27.9%	-1.2	ref		544	38.1%	-1.1	ref	
2^nd^ Quartile	715	25.7%	-3.4	.894		496	37.1%	-2.1	.959	
3^rd^ Quartile	696	31.7%	+2.6	1.198		530	41.4%	+2.2	1.50	
4^th^ Quartile	852	31.2%	+2.1	1.170		667	39.8%	+0.6	1.076	
Agreeable	3008				X^2^(3) = 8.4	2237				X^2^(3) = 6.4
1^st^ Quartile	733	25.1%	-4.0	ref		518	35.3%	-3.9	ref	
2^nd^ Quartile	699	31.3%	+2.2	1.363		511	42.8%	+3.6	1.374	
3^rd^ Quartile	636	29.4%	+0.3	1.244		485	38.5%	-0.7	1.152	
4^th^ Quartile	940	30.6%	+1.5	1.319		723	40.0%	+0.8	1.223	
Conscientious	3008				X^2^(3) = 12.8	2237				X^2^(3) = 5.0
1^st^ Quartile	707	24.7%	-4.4	ref		483	36.1%	-3.1	ref	
2^nd^ Quartile	691	31.6%	+2.5	1.410		530	41.2%	+2.0	1.240	
3^rd^ Quartile	818	28.0%	-1.1	1.186		610	37.6%	-1.6	1.066	
4^th^ Quartile	792	32.2%	+3.1	1.447		614	41.6%	+2.4	1.265	
Neuroticism	3008				X^2^(3) = 10.7	2237				X^2^(3) = 6.7
1^st^ Quartile	671	34.2%	+5.1	ref		523	43.9%	+4.7	ref	
2^nd^ Quartile	819	28.4%	-0.7	.763		601	38.7%	-0.5	.807	
3^rd^ Quartile	681	27.3%	-1.8	.722		502	36.9%	-2.3	.748	
4^th^ Quartile	837	27.4%	-1.7	.728		611	37.5%	-1.7	.767	
Openness	3008				X^2^(3) = 10.4	2237				X^2^(3) = 6.3
1^st^ Quartile	746	28.5%	-0.6	ref		537	39.5%	+0.3	ref	
2^nd^ Quartile	689	24.8%	-4.3	.828		494	34.5%	-4.7	.807	
3^rd^ Quartile	814	30.8%	+1.7	1.120		621	40.4%	+1.2	1.034	
4^th^ Quartile	759	32.0%	+2.9	1.179		585	41.6%	+2.4	1.089	
Life Satisfaction	3009				X^2^(2) = 10.4	2237				X^2^(2) = 0.9
Low	258	23.6%	-5.5	ref		168	36.5%	-2.7	ref	
Moderate	1061	27.8%	-1.3	1.24		763	38.6%	-0.6	1.093	
High	1690	30.8%	+1.7	1.44		1306	39.9%	+0.7	1.155	
Cantril Ladder	1708				X^2^(2) = 7.4	1216				X^2^(2) = 1.1
Low	173	25.0%	-4.1	ref		106	40.7%	+1.5	ref	
Moderate	793	31.2%	+2.1	1.36		544	45.4%	+6.2	1.213	
High	742	35.2%	+6.1	1.63		566	46.2%	+7.0	1.254	
Global Health	1221				X^2^(2) = 18.2***	848				X^2^(2) = 2.8
Excellent	596	37.0%	+7.9	ref		453	48.7%	+9.5	ref	
Moderate	413	31.5%	+2.4	.782		283	45.9%	+6.7	0.894	
Poor	212	20.9%	-1.0	.448		112	40.0%	+0.8	0.702	

*** significant at p < .0026

Note: Values are adjusted for invitation month of entry; panelists whose participation status was undetermined at the time of analyses were omitted.

### Personality assessment

The 44-item Big Five Inventory [42] was used to assess panelists’ personality characteristics on the following dimensions: Extraversion, neuroticism, conscientiousness, agreeableness, and openness to experience. The Big Five Inventory was administered repeatedly in 2-year intervals. The personality assessment included in this analysis was from the latest personality assessment that the invited panelists completed prior to when the EMA burst study invitation was made available to them.

### Analysis plan

A total of 3,169 UAS panelists were invited to participate in the EMA study. For each of the (primary and exploratory) research questions we present the uptake (i.e., participation) rate based on the definitions of the groups being compared. To evaluate selection bias, we conducted logistic regressions wherein each selection variable was separately used to predict dichotomous participation status. A categorical control variable was also included in these models to remove variance associated with the month participants were invited into the study. This adjustment seemed especially prudent given that data were collected in 2020, the year of the COVID-19 pandemic, and pandemic-related factors, such as stay-at-home orders and employment changes, could have affected participation over time.

Another consideration for the analyses was that at the time when analyses were run the final classification status of 155 individuals was not known. These individuals had been contacted, yet the study staff did not yet know if they would enter the momentary study. These individuals were therefore excluded from all analyses, reducing the final analytic sample to 3,014.

Logistic regression results are presented together with estimated percent uptake rates for each of the categories of the selection variables, after month-of-invitation was controlled. For the continuous age variable, the distribution was split into six categories corresponding to ten-year blocks (except for the lowest and high categories). For the continuous personality variables, quartiles were used to define the categories, based on the distribution of the variable for the available sample size. To create meaningful categories with reasonable Ns, the categories of some of the variables were collapsed. Variables collapsed into three categories were education (less than high school, high school, college), race (White, Black, other race), and prior experience with UAS surveys (lowest & low, high, highest), life satisfaction and the Cantril Ladder (low [0–4), moderate [5–7], high [8–10]), global health (excellent [1–2], moderate [3], poor [4–5]), and household income was collapsed into four ordinal categories (<$30,000, $30,000-$59,999; $60,000-$99,999; = >$100,000). Considering that there were 19 selection variables in these analyses (for each of the research questions), a Bonferroni correction was applied to the significance level for each set of univariate analyses yielding a significance threshold of .0026.

It is likely that there was overlap (collinearity) among the selection variables. We examined the associations between all 19 predictor variables and report associations at least of moderate magnitude, by convention. We report associations with a Cramer’s V value of at least .40.

The final steps of the analyses were to create an uptake prediction model using all of the selection variables entered simultaneously as predictor variables in a multiple logistic regression model. This regression analysis shows which variables contributed uniquely to the prediction of who participated in the study. Using this model, it was possible to estimate uptake for hypothetical individuals with any set of levels on the selection variables, which allowed us to illustrate the magnitude of the impact of selection variables on uptake.

For the first exploratory analysis we tested possible interactions among pairs of selection variables that emerged as significant from the multiple regression model, where a separate regression was used for each of the variable pairs. We also check for specification errors using STATA’s “linktest” command. Finally, in the second two exploratory analyses we created a variable for individuals who consented to participate in the study where 0 indicates that they did not complete any EMA prompts (“dropped out”) and 1 indicates that they entered the EMA protocol and completed prompts. Another variable extended the definition of drop-out to any participant who completed less than half of the scheduled prompts. The set of predictors described above was used to predict these dichotomous variables, controlling for the month-of-invitation. All analyses were conducted in STATA16.

## Results

### Classification of participation status

**Fig 1:**
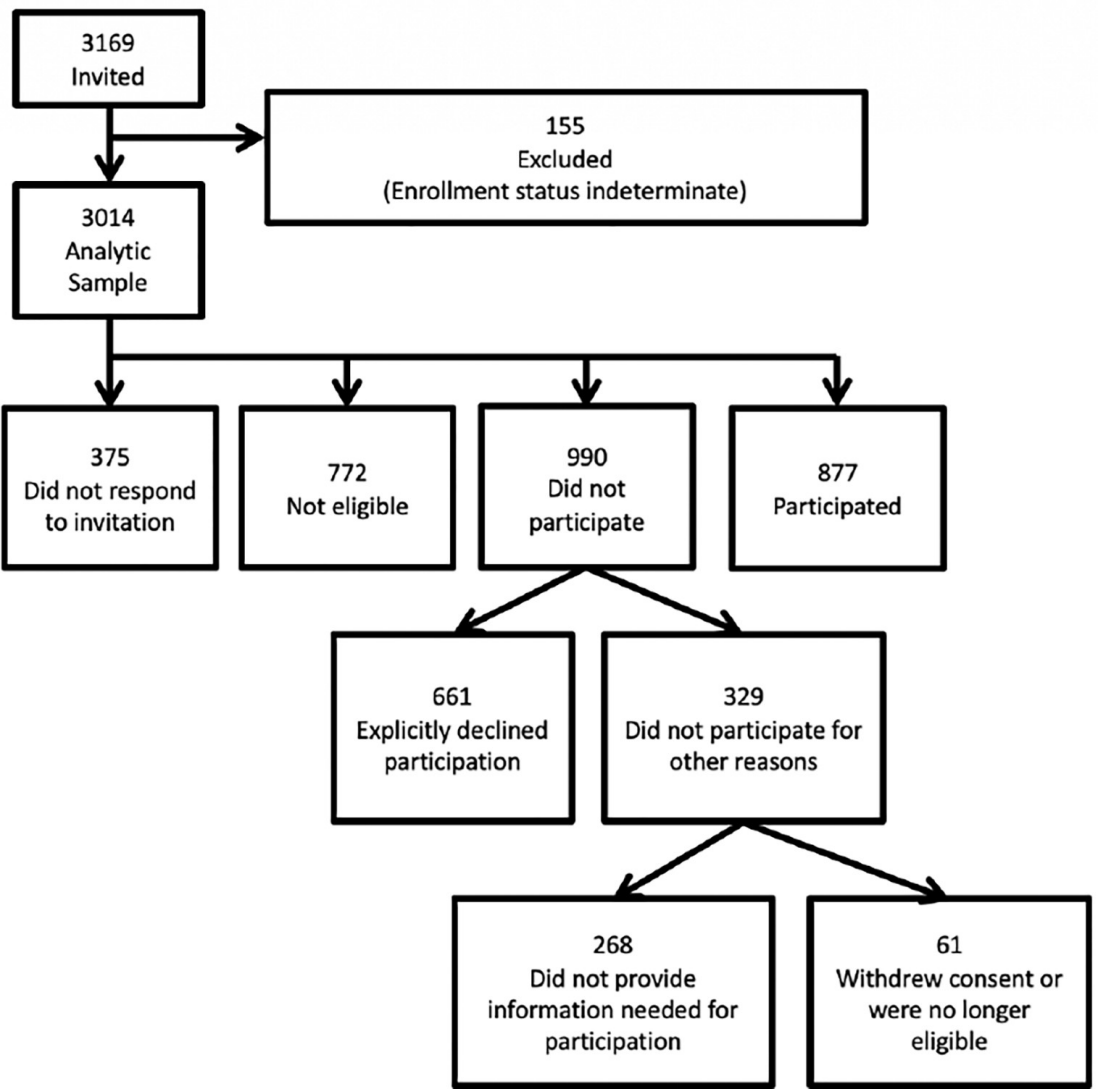
Study recruitment flow.

To clarify how respondents were classified into the different groups used for analysis, Fig 1 presents a flowchart of the various pathways an individual could take resulting in their final classification. These pathways naturally led to the definitions of participants versus nonparticipants as addressed in the two primary questions. Almost all individuals who chose to participate did so in response to the first invitation.

### Primary Question 1: Are those who participated in the momentary study different from those who did not?

This analysis contrasted participants and all others in the sample. There were 877 individuals whose final categorization was as “participated” and 2,137 as “did not participate,” yielding an overall uptake rate of 29.1%. Participation was defined as entering the momentary burst study and was not dependent upon how many momentary assessments a participant actually completed during the burst. Results from logistic regressions of the selection variables are presented in Table 1 (left section). The 3^rd^ column of the table presents the estimated uptake rate for each category of the selection variable. This is followed by the difference between that category and the average for the entire group (i.e., 29.1%) to facilitate interpretation. The next two columns show the odds ratio for each category and the overall significance for the selection variable.

Month of invitation, the covariate in the analysis, was not significant in any of the univariate regression models but several selection variables were significantly associated with participation. Compared with participants, those who did not participate–as broadly defined for this analysis–were more likely to be older, male, to have less education and lower income, not working, retired, disabled, to have less confidence in their computer keyboard and general computer skills, to report having taken fewer UAS surveys, and who have worse self-reported health. Many of the effects were of substantial magnitude, as can be discerned from the difference in uptake rates presented in the table for each level of the variables. For example, uptake was twice as high in those with college education compared with those with less than a high school education; the same was true for those with high and low incomes. Uptake for those reporting high confidence in their computer skills was quadruple the rate of those with low confidence.

### Primary Question 2: Of individuals who were deemed eligible, are those who participated different from those who did not?

As previously mentioned, many individuals were not presented with the study description, because they did not meet eligibility requirements (n = 773 individuals). Thus, they did not have the opportunity to accept or decline the invitation. The prior analyses viewed ineligible panelists as nonparticipants; this analysis omits ineligible individuals. Month of invitation, the covariate in the regression, was not significant. Overall, the uptake rate for this comparison is 39.2%, about 10% higher than the rate from the previous analysis. Results for the prediction of participation status from the selection variables are shown in Table 1 (right section). The findings are very similar to those in Question 1, with the exception that the variable *disabled* is no longer significant.

### Predictive model based on all selection variables for Question 1

This analysis addressed the question, what would be the predictive model of participation (as defined for Question 1) if a researcher had all 22 selection variables in hand? The first logistic regression model included all predictor variables of participation versus nonparticipation simultaneously, but because of the missing values on some selection variables, the total sample size for this analysis was only 968. The overall regression model was significant (X^2^(51) = 200.6, p < .000) with a McFadden’s pseudo R^2^ of 16.4%. In this model, those who did not participate were more likely to be older, male, to have lower income, and to have less confidence in their computer keyboard skills than participants. Given the first model’s reduced N, a second regression was run that dropped the variables with low Ns (the two computer savvy variables, Cantril ladder, and global heath). The second regression replicated the results of the first for those variables in the model.

We next estimated the uptake probability for those panelists scoring in the least favorable category for each of the four significant uptake variables (that is, using the categories that were associated with the lowest uptake rate) and the uptake probability for those in the most favorable category by substituting values into the regression equation. The uptake rate for the “worst case” panelist was estimated at 2% (that is, someone over age 75, male, income under $30,000, and who was not confident in their computer skills) and for the “best-case” panelist uptake was estimated at 66% (under age 55, female, income greater than $100,000, and completely confident in their keyboard skills).

### Interactions among unique selection variable predictors

All prior analyses considered only the main effects of selection variables on uptake, but it is plausible that there are interactive effects among the predictor variables. To explore this possibility, we tested interactions among all unique pairings of the selection variables that were significant in the previous multiple regression analysis (predicting participation as defined for Question 1). There were 6 pairs of interaction terms created by these 4 variables. None of the tests for interactive effects was significant at the .0026 level. The second test was for specification errors using the full model (with all predictor variables) and the lack of a significant linktest result suggested that interactions among predictors would not improve model fit.

### Collinearity among selection variables

Few associations among the selection variables exceeded the a priori selected Cramer V threshold of .40: being retired was associated with higher age (V = .67); a higher likelihood of working was also associated with higher age (V = .48) and with being retired (V = .64); the two computer skills variables were also positively associated (V = .45); and, life satisfaction was associated with the Cantril ladder (V = .43). Overall, this suggests that multicollinearity among the predictors was not a serious concern in the analyses.

### Exploratory Question 1: Were those who explicitly declined participation different from those who participated?

We next contrasted those who explicitly informed us that they were not interested in participation (which implies that they were eligible for the study, see Fig 1) with those who participated. Month of the invitation was associated with a higher rate of saying “No” in the later part of the year, as was being older, being male, and being retired. Being less computer savvy was also associated with a higher chance of saying No, as was reporting previously participating in fewer UAS surveys. These findings were very similar to those for the prior analyses. However, income, education, disability, working, and having poorer health no longer met the significance threshold.

### Exploratory Question 2: Prediction of dropouts

Of the 877 individuals who consented to participate in the EMA study, 193 (22%) completed no EMA prompts, and an additional 55 individuals (6.2% of total) completed less than half of the scheduled prompts. To control for month-of-invitation in the prediction of dropping out, the categories of invited-at-months 5, 8, and 10 were collapsed into a single category, because there were only 2 individuals who dropped in those months. Most of the dropouts occurred in months 2 and 3.

None of the explanatory variables were significant predictors of dropout, defined as either completing no EMA prompts or as less than half of the scheduled prompts at or beyond the adjusted alpha level of .0026. Given that these are exploratory analyses, we report two variables that would have met the .05 alpha level. When dropout was defined as completing no prompts, working was associated with a slightly higher dropout rate (.76) versus those who reported not working (.79, p = .011) and being retired was associated with higher a dropout rate (.77) than not being retired (.79, p = .028). Analyses including as drop-outs those who completed less than half of the scheduled prompts revealed no associations with the predictor variables.

## Discussion

Using an Internet panel that allowed us to characterize both participants (those who consented to participate) and nonparticipants who were offered participation in a momentary data collection study, the broad goals of this investigation were to determine the uptake rate for the sample and to examine possible participant selection differences between those who participated and those who did not. First, uptake rates are of interest because so little is currently known about what proportion of individuals who are invited into momentary studies consent to participate in them. This is largely due to the lack of knowledge about the population from which individuals are sampled (e.g., when EMA studies use posted flyers for recruitment of or when media advertisements are used for solicitation). Without knowing the number of people who read a flyer or were approached with the invitation, it is not possible to estimate likely uptake rates. Second, we examined differences between participants and nonparticipants to investigate factors associated with participant selection bias. Such information is necessary to understand the generalizability of studies’ results to other participants and settings, that is, external validity. We also examined potential differences between those who agreed to participate, but did not complete any EMA prompts (dropout) versus those who completed prompts.

### Uptake rates in EMA research

We first consider uptake rates for the two primary questions, which differed in the way individuals who were deemed ineligible for the study were treated. For the first question, ineligible individuals were included in the computation of nonparticipants resulting in an uptake rate of 29.1%. On one hand, we view this rate as rather impressive given our *a priori* expectation that uptake would be considerably lower. On the other hand, however, in considering the magnitude of the uptake rate, we cannot lose sight of the fact that this study involves multi-stage sampling: the population of the UAS panel was previously selected from a larger population and the uptake rate for being a panelist in the UAS in the first place was about 12.4%. Therefore, UAS panelists can already be considered cooperative individuals. With this in mind, perhaps a more prudent way of thinking about the 29.1% uptake is to revise the figure by multiplying it by the UAS uptake rate, which yields an adjusted rate of 3.6%. From this revised perspective, uptake was low and consistent with our expectations. This conclusion, however, is tempered by the fact that we do not know if the sample invited into the UAS panel would have had the low uptake rate had the invitation been for a momentary study. We also need to keep in mind that the rates are based on an older sample of individuals (50 years of age and older) and many EMA studies employ students as participants. These considerations suggest additional research on this question with a sample with a wider age range.

For the second primary question, where ineligible participants were eliminated from the pool of nonparticipants, the uptake rate increased by 10 points to 39.2%. Taking into account the participation rate for the panel, the estimated adjusted uptake for this group is about 5%. Again, given the lack of prior work on uptake rates, this seems a plausible, yet rather low, magnitude for uptake to a momentary study requiring use of a smartphone. The observed increase in update is certainly sensible and perhaps is better estimate of uptake, because arbitrary (though understandable) study-specific eligibility factors are reduced.

### Factors associated with selection bias in EMA

Turning to the second focus of this paper–participant selection bias–there was strong evidence that the levels of the selection variables varied between those people who participated in the momentary study compared to those who did not. These differences were quite similar in the two primary analyses. Within the scope of recruitment from this particular Internet panel, many person characteristics previously shown to increase study uptake for research studies in general (i.e., apart from EMA studies) were replicated here. The “younger” individuals in this sample (remembering the age of this sample) had a considerably higher participation than those who were older. Likewise, females were 30–35% more likely to participate than males. Individuals with college education were much more likely to participate than those with less than a high school education and, those with high incomes had greater uptake. Being employed and not being retired, two closely related variables, were also predictive of high uptake rates. Furthermore, those who rated their health as excellent were almost twice as likely to participate as those in the lower health categories [43]. In terms of the magnitude of effects, we view the resulting selection biases as substantial and they should give pause to any momentary researchers who believe that their findings generalize to the population from which the sample was drawn.

Also consistent across the analyses were the remarkable associations that the two computer skills had with uptake rates. Those who said they did not have confidence in their computer skills (even though they had agreed to answers surveys on-line) had an uptake rate of only 11% whereas the much larger group who rated their confidence in the most skilled two categories had an uptake rate of 43%, almost a fourfold difference (for Question 1). Although skills with computers are likely associated with smartphone skills, this may not be a strong association (i.e., some people could have poor computer skills yet be competent with their smartphones). Smartphone skills may be a better predictor of uptake in EMA studies than computer skills, and should probably be included in future studies of selection. These results are evidence of participant selection bias and may be actionable in future momentary studies. For example, highlighting the fact that thorough training of prospective participants in the technology (live training or extensive web-based video training) would be available, and making the technology more accessible via simplified devices (say, dedicated for the collection of momentary data), could make the study more attractive to those with less technology skill.

Yet another strong predictor of uptake was the number of prior UAS surveys individuals reported participating in previously. Although this variable may be uniquely available in panel studies with their repeated measurements (as in the UAS), it nevertheless goes to the importance of respondent motivation. High level of participation in past surveys probably implies a high motivation, although the reasons for the motivation may differ widely across respondents. Our findings suggest that people who do not participate in momentary studies may have less survey experience of all kinds, providing further evidence that selection bias is present.

Although we thought it plausible that selection variables would interact with one another, among the four variables with strongest predictive ability in the simultaneous multiple regression model, no interactions were detected. It is possible that a more comprehensive testing of all 19 variables interacting in pairs or triplets could have shown effects, but we did not pursue this route given the extremely large number of tests that would result and possibility of detecting chance associations. We discuss the implications of the findings below.

Regarding the first exploratory question that examined those who explicitly declined participation, the results were generally consistent with the primary analyses, although some variables that were significant in those analyses were no longer significant. In part, this may be the result of lower statistical power due the reduced sample size for these regressions (and the corrected alpha levels used). We did not see strong evidence to suggest that explicitly saying No to the invitation to participate in the momentary study was associated with any unique participant characteristics over those identified in the primary analyses.

The second exploratory question went beyond the primary question of uptake, as defined by consenting to participation, and asked whether or not those who consented, but did not provide EMA data, were different than those who did provide data. A substantial proportion of those who consented did not provide EMA data, and most of these instances happened early on in the recruitment period (the first 3 months). None of the predictor variables were significantly associated with providing EMA data, and there was only a suggestion that not being retired and working were associated with providing data (only a 2–3% difference). One possible explanation for these results is that the reasons for not providing data were attributable to operational difficulties in the early stages of the study (e.g., technological challenges) and had little to do with person characteristics (hence, no selection bias was detected).

### Strengths and limitations of this study

Before turning to other implications of these results, we discuss the strengths and limitations of the study. As mentioned in the introduction, we recognize the unique setting for this project and the advantage it conferred by having available much information about nonparticipants. But most momentary research will not be conducted with Internet panels, so the generalizability of the findings to other samples and settings should be considered limited. Additionally, even though the UAS panel covers the full adult age range, invitations to participate in the EMA study were limited to panelists aged 50 years and older, further reducing generalizability. Although the method for recruiting individuals to the UAS was based on representative sampling techniques and yielded a probability panel, that 12% of those approached became panel members suggests the potential for selection bias at the panel recruitment stage.

Furthermore, we do not know the degree to which these results generalize to other EMA designs. This study used electronically administered EMA, was focused on particular content areas, prompted respondents 6 times a day, had assessments of a particular duration, and asked for 7 days of participation. We do not know if the same results would be found, for example, for paper-and-pencil versions of EMA, for studies concerned with other content areas, for studies with fewer or greater numbers of daily prompts, for those with shorter or longer assessments, and, finally, for studies requiring a fewer or greater number of reporting days. There is some suggestion that these factors matter, but their effects are small [13]. Parametric studies of the factors just mentioned would be very welcome, although such research would be resource intensive.

It may also be the case that UAS panelists were *already* selected into the panel by factors that were identified in this study. For instance, we found strong selection bias based on two variables tapping familiarity and comfort with computers. We think it plausible that UAS recruitment procedures had already excluded many individuals not comfortable with computers, and that would have the effect of enhancing uptake rates in the current results (because the sample invited to participate in the burst may have been skewed toward computer savvy individuals). This conclusion is speculative and confirmation of these findings with non-panel samples is needed. We further note that our focus here has been on descriptive statistics of those who did and did not participate in this EMA study. That is, we found evidence of selection bias in selected characteristics of participants we examined. We did not explore the effect of selection bias on associations between variables. It is possible that associations among variables that are the focus of momentary measurement are unaffected by the types of biases we have identified.

### Implications for EMA research

To place the results into perspective, if we had observed that everyone who was offered participation agreed by consenting, then there would be less concern about selection bias (of course, assuming appropriate sampling procedures). This was far from the case in the work reported here, where in even in the most optimistic case only between 29% and 39% of the sample agreed to participate. This implies a high probability of selection bias as people were self-selecting into the study, and a distinct possibility of untoward effects on findings regarding how well they represent the broader population from which they were drawn. If we consider the estimated rate of uptake from a general population–as opposed to from the panel, which was 5% or less–then the likelihood of selection bias increases considerably and may be of notable concern.

We believe that the variables identified in this study–even taking into account the caveats associated with recruiting from an existing Internet panel–are useful for understanding at least some of the factors associated with volunteering for a momentary data collection study. Based on these results, selection bias would be likely to yield a particular composition of participants who are more likely to be women, have higher incomes, be better educated, be working, be healthy, and be comfortable with computers. As discussed in the introductory material, the particular impact of these selection results on the generalizability of observed associations to the population will depend on the nature of the association being studied and how those variables might distort that association (relative to the associations in other samples).

Admittedly, this requires a thoughtful appraisal of such possibilities, because it is unlikely a researcher would know the answer to the question with much confidence. For instance, if a study had the goal of describing the association between momentary stressors and affect and if the recruitment was dependent upon flyers or advertisements (where self-selection is inherent), then one would have to evaluate the possibility that the association observed for a more female, better educated, higher income, working, healthy, and computer savvy group of participants would be different from the association for the population from which the (biased) sample was drawn. Judgments like these are not at all straightforward, but are a necessary component of evaluating threats from participant selection bias. Our results suggests that it would be prudent to seriously consider such questions when reporting research findings.

Nevertheless, we can imagine how at least one of the findings reported here could inform a revision of the invitation for EMA studies. People with lower levels of technical skills (keyboard) and lower confidence in their computer skills were less likely to participate. Perhaps a study invitation saying that no special computer or phone skills were required for participation and that procedures would be clearly explained could increase uptake for those with low skills or low confidence. Another possibility for “countering” the tendency for non-working and retired individuals to participate at lower rates would be to increase monetary incentives (if allowable by institutional review boards) with the hope that this would differentially increase interest in those with (presumably) lower incomes. Regarding increasing the appeal of EMA studies to those in relatively poor health, perhaps emphasizing the short amount of time required to complete an EMA assessment would reassure them that they could successfully complete the study protocol. Of course, these are speculations that require empirical confirmation.

Another implication of these results concerns the usefulness of shifting EMA studies from small volunteer or “captive” samples to population-representative samples where inferences are presumed to be applicable to many more people. As we have demonstrated in this paper, conducting EMA research within the scope of existing probability panels provides much needed information about the characteristics of individuals from which the sample was drawn. At the same time, the selection biases identified here indicate the kinds of challenges that may be inherent in achieving high levels of recruitment in population samples that could lead to universal inferences about people’s experiences in their natural daily environments, rather than inferences restricted to highly select samples (such as those discussed in the WEIRD argument).

In summary, this study adds to our knowledge about the rate of uptake into momentary studies and about factors that make it more likely that people will volunteer for such studies. Admittedly, this an initial and imperfect approach to addressing the question, and we strongly encourage continued exploration into this important methodological feature of momentary research.

## Supporting information

S1 Appendix (DOCX)
