# Supplementary material for: Shedding light on participant selection bias in Ecological Momentary Assessment (EMA) studies: Findings from an internet panel study
Source: PLoS One. 2023 Mar 9;18(3):e0282591. doi: 10.1371/journal.pone.0282591 (PMC9997985; doi:10.1371/journal.pone.0282591)
Supplement: S1 Appendix — (DOCX) [file pone.0282591.s001.docx]

**Appendix 1: Text of email invitation sent to potential participants.**

Subject: Scheduling your UAS 7-Day Project – please respond

Dear [UAS member]

Thank you for agreeing to participate in the Understanding America Study’s 7-Day Tracking Your Daily Life Project.

**Please respond to this email right away, as we have a 7-day project starting very soon.**

**1. As a reminder, here is what you will do during your project week:**

- Answer prompts from the phone app six random times every day
- Log into your UAS page each evening to complete a short survey
- Two days out of the week, you will be prompted to make a short recording in the UAS EMA app
- To earn the full $100 reward for the project**,** you will complete all of these tasks every day.

NOTE: Please check your schedule to make sure that you are able to spend 2 minutes answering survey questions at six random times every day of your scheduled week. If another week would work better for you, let us know!

**2. Next project dates: [start_date] through [end_date]. Are you able to participate?**

**Please respond by [deadline_date] and let us know if you are available or if you would prefer to schedule for a different week.**

If you have questions about the project, review your Project Information Sheet or contact the UAS helpdesk.

Thanks again! We appreciate your participation!

[staffmember_name]

Understanding America Study (UAS)

Helpline: [helpline_number]

email: [helpline_email]
